# Data in support of comparative proteomics analysis of superior and inferior spikelets in hybrid rice during grain filling and response of inferior spikelets to drought stress using isobaric tags for relative and absolute quantification

**DOI:** 10.1016/j.dib.2014.08.001

**Published:** 2014-08-12

**Authors:** Minghui Dong, Junrong Gu, Li Zhang, Peifeng Chen, Tengfei Liu, Jinhua Deng, Haoqian Lu, Liyu Han, Buhong Zhao

**Affiliations:** Suzhou Academy of Agricultural Science, Suzhou, 215155, PR China

## Abstract

We provide the raw data for protein and peptide identification and quantization of superior and inferior spikelets in hybrid rice during grain filling. The mass spectrometry proteomics data have been deposited to the Proteome Xchange Consortium via the PRIDE partner repository with the dataset identifier PXD001046. Our data presented here is also related to the article “Comparative proteomics analysis of superior and inferior spikelets in hybrid rice during grain filling and response of inferior spikelets to drought stress using isobaric tags for relative and absolute quantification ”in the Journal of Proteomics [1].

## Specifications table

**Table:** 

Subject area	Biology

More specific subject area	Rice cultivation
Type of data	Table, excel files
How data was acquired	iTRAQ labeling (Applied Biosystems)
	SCX chromatography and AKTA Purifier system (GE Healthcare)
	Q-Exactive MS (Thermo Finnigan) and Easy nLC (Proxeon Biosystems, now Thermo Fisher Scientific)
	MASCOT engine (Matrix Science, London, UK; version 2.2)
	Rice sequence database (uniprot_*Oryza_sativa*.fasta, released in February 2013, 144512 sequences)
Data format	Raw and analyzed
Experimental factors	Panicles that headed on the same day were chosen and tagged, and the flowering date of each spikelet on the tagged panicles was recorded. Fifteen tagged panicles were sampled at 7 days and 14 days after anthesis. Superior spikelets (SS) and inferior spikelets (IS) were collected, in addition, three irrigation patterns were set from c, i.e. shallow water irrigation (water status was controlled at 0 kPa), light wetting-drying irrigation (water status was controlled at −20 kPa), and heavy wetting-drying irrigation (water status was controlled at −40 kPa).
Experimental features	Isobaric tags for relative and absolute quantification (iTRAQ)
Data source location	Suzhou, China
Data accessibility	The mass spectrometry proteomics data have been deposited to the Proteome Xchange Consortium (http://www.proteomexchange.org/) via the PRIDE partner repository with the dataset identifier PXD001046.
	Other datasets are directly provided with this article.

## Value of the data

• The data including the raw data of protein and peptide identification and quantization can be reused by other scientists investigating hybrid rice under various conditions.
• The bioinformatics data can provides insight into the biological function of the successfully identified proteins.

## 1 Experimental design, materials and methods

Panicles that headed on the same day were chosen and tagged, and the flowering date of each spikelet on the tagged panicles was recorded. Fifteen tagged panicles were sampled at 7 days and 14 days after anthesis. Superior spikelets (SS) and inferior spikelets (IS) were collected, in addition, three irrigation patterns were set from c, i.e. shallow water irrigation (water status was controlled at 0 kPa), light wetting-drying irrigation (water status was controlled at −20 kPa), and heavy wetting-drying irrigation (water status was controlled at −40 kPa).

### 1.1 Rice cultivation

Field experiments were carried out in an experimental farm of Taihu Area Institute of Agricultural Sciences, Su Zhou, Jiangsu province, China in 2011, with large-panicle hybrid Japonica rice Yongyou 8 (according to the test for an average of 181 grains with 26.3 g weight per panicle) as the material. Seedlings were sown on 20th May and transplanted on 25th June at a hill spacing of 0.3 m×0.15 m with 1 seedling per hill. The soil of the field was paddy soil that contained 2.42% organic matter and 158.4, 8.4 and 127.0 mg kg^−1^ available N–P–K respectively. Field management was in accordance with the conventional technique for high-yield cultivation, N fertilizer (225 kg/hm2), basal-tiller N fertilizer to ear-grain N fertilizer (6:4), the basal to ear-grain N was 2:1, and ear-grain N fertilizer was used when the last fourth or fifth leaves came out. P fertilizer was converted into P_2_O_5_ (70 kg/hm2) as the basal fertilizer and K fertilizer was converted into K_2_O (150 kg/hm^2^) according to the ratio of basal-tiller N fertilizer to ear-grain N fertilizer (5:5).

### 1.2 Collection of superior and inferior spikelets

Panicles that headed on the same day were chosen and tagged, and the flowering date of each spikelet on the tagged panicles was recorded. Two hundred panicles that headed on a same day were tagged. The flowering date and position of each spikelet on the tagged panicles were recorded. Fifteen tagged panicles were sampled at 7 days and 14 days after anthesis (DAA, the day was accounted from the first day after flowering). Superior spikelets (SS) and inferior spikelets (IS) were collected according to the previous report [2], then were frozen in liquid N_2_ and then stored at −70 °C for protein extraction.

### 1.3 Water stress treatment

Three irrigation patterns were set from c, i.e. shallow water irrigation (water status was controlled at 0 kPa), light wetting-drying irrigation (water status was controlled at −20 kPa), and heavy wetting-drying irrigation (water status was controlled at −40 kPa). The test base was covered by weather shed. The water status was determined at 7:00–8:00 and 16:00–17:00 every day by a portable digital measuring instrument for soil water potential and temperature (TRS-II, Zhejiang Tuopu Equipment Co., Ltd.). Shallow water irrigation was performed when the water status was lower than the set value.

### 1.4 Protein extraction and digestion

Frozen rice tissue was finely powdered in liquid nitrogen, and precipitated for 1 h with 25 ml TCA/acetone (1:9, containing 65 mM DTT) at −20 °C. The homogenate was centrifuged and the pellets were air-dried, dissolved in 30 μL STD buffer (4% SDS, 150 mM Tris–HCl, pH 8.0), incubated with boiling water for 5 min, cooled to room temperature, and diluted with 200 μL of UA buffer (8 M urea, 150 mM Tris–HCl, pH 8.0). The homogenate was centrifuged, the supernatants were collected and the protein content was determined by a BCA protein assay reagent (Table 1).

The retained protein was washed with 200 μL of UA buffer, centrifuged, and added with 100 μL of UA buffer containing 0.05 M iodoacetamide. The mix was incubated for 20 min in dark and then centrifuged under the above conditions. The filter was then washed three times with 100 μL of UA buffer, 100 μL of DS buffer (50 mM triethylammonium bicarbonate, pH 8.5) was added. Then the solution was centrifuged for 10 min in the same condition. This step was repeated twice. Finally, 40 μL of DS buffer containing 3 μg trypsin (Promega) was added to each filter. The samples were incubated overnight at 37 °C, and the resulting peptides were collected by centrifugation. The peptide content was estimated by UV density at 280 nm.

### 1.5 iTRAQ reagent labeling and liquid chromatography (LC)

iTRAQ labeling was performed according to the manufacturer׳s instructions (Applied Biosystems). Briefly, the peptide mixtures were reconstituted with 30 μL of iTRAQ dissolution buffer. The label method of every sample (45 μg) using iTRAQ Reagent-8plex Multiplex Kit (AB SCIEX) is shown in Table 1, and every sample was labeled twice. The aliquots of iTRAQ were combined with peptide mixtures from 5 different samples, respectively, and incubated at room temperature for 1 h. REF was a mixture containing same-amount proteins of the five samples.

Prior to LC-MS/MS analysis, the peptides were purified to eliminate excess labeling reagent by SCX chromatography using an AKTA Purifier system (GE Healthcare). A 10 μL solution from each peptide fraction was injected for nanoLC-MS/MS analysis using a Q-Exactive MS (Thermo Finnigan) equipped with Easy nLC (Proxeon Biosystems, now Thermo Fisher Scientific). The peptide mixture (5 μg) was loaded onto a C18-reversed phase column packed in-house with RP-C18 resin (5 μm) in buffer A (0.1% formic acid) and separated with a linear gradient of buffer B (0.1% formic acid in 80% acetonitrile) at a flow rate of 250 nL/min controlled by IntelliFlow technology over 140 min.

### 1.6 Electrospray Ionization (ESI) tandem MS (MS/MS) analysis by Q exactive

MS data were acquired using a data-dependent top10 method dynamically choosing the most abundant precursor ions from the survey scan (300–1800 m/z) for the HCD fragmentation. The target value was determined based on predictive Automatic Gain Control (pAGC). The dynamic exclusion duration was 60 s. Survey scans were acquired at a resolution of 70,000 at m/z 200, and resolution for the HCD spectra was set to 17,500 at m/z 200. The normalized collision energy was 30 eV, and the underfill ratio, which specifies the minimum percentage of the target value likely to be reached at maximum fill time, was defined as 0.1%. The instrument was run with peptide recognition mode enabled (Supplementary table 2).

### 1.7 Sequence database searching and data analysis

MS/MS spectra were searched using MASCOT engine (Matrix Science, London, UK; version 2.2) against a rice sequence database (uniprot_Oryza_sativa.fasta, released in February 2013, 144512 sequences). The MASCOT search results were further processed using ProteomicsTools (version 3.05). Assembling protein identifications were qualitatively analyzed by Proteome Discoverer1.4 software. All data were reported based on 99% confidence for protein identification as determined by false discovery rate (FDR) ≤1%. Isobaric Labeling Multiple File Distiller and Identified Protein iTRAQ Statistic Builder were used to calculate the ratios of protein, in which Sample REF was used as the reference, based on the weighted average of the intensities of report ions in each identified peptide (See Supplementary table 1). The final ratios were then normalized with the median average protein ratio, assuming that most proteins remained unchanged in abundance. Only the protein identification that was inferred from the unique peptide identification in two independent experiments was considered. Statistical analysis was conducted using a one-way ANOVA. P-values≤0.05 by Tukey׳s test were considered significant. Among the statistically significant proteins detected by the ANOVA test (p<0.05), proteins abundances that changed less than 1.5-fold or 1.2-fold were discarded (See Supplementary table 4).

### 1.8 Bioinformatics analysis of the differentially abundant proteins

Sequence data of the selected the differentially abundant proteins were retrieved from UniProtKB database (Release 2013_07) in batches in FASTA format. The retrieved sequences were locally searched against Swiss-Prot database (plant) using the NCBI BLAST+ client software (ncbi-blast-2.2.28±win32.exe) to find homolog sequences from which the functional annotation was transferred to the studied sequences. In this study, the top 10 blast hits with E-value less than 1e−3 for each query sequence were retrieved and loaded into Blast2GO (Version 2.6.6) for Gene Ontology (GO) mapping and annotation. The sequences without BLAST hits and the un-annotated ones were then selected to go through InterProScan against EBI databases to retrieve the functional annotations. The GO project described the roles of proteins in three domains: biological process, molecular function and cellular component. Following annotation and annotation augmentation, enzyme codes were sequentially mapped to annotated sequences and metabolic pathways in Kyoto Encyclopedia of Genes and Genomes (KEGG, http://www.genome.jp/kegg/) [3] (Supplementary table 3).

## Figures and Tables

**Table 1:** Protein contents of inferior spikelets 8 DAA under 0 kPa (IS8DAA, A), superior spikeletes on 8 DAA (B, SS8DAA) under 0 kPa, and inferior spikelets on 15 DAA under 0 kPa (C, IS15DSS), −20 kPa (D) or −40 kPa (E).

Sample	A	B	C	D	E
Protein content (μg/μL)	7.86	8.12	8.25	7.24	7.40

## References

[1] Comparative proteomics analysis of superior and inferior spikelets in hybrid rice during grain filling and response of inferior spikelets to drought stress using isobaric tags for relative and absolute quantification, J. Prot., in press 10.1016/j.jprot.2014.07.001 25058577

[2] Dong M.-h., Chen P.-f., Xie Y.-l., Qiao Z.-y., Yang J.-c. (2012). Variations in carbohydrate and protein accumulation among spikelets at different positions within a panicle during rice grain filling. Rice Sci..

[3] Kanehisa M., Goto S., Sato Y., Furumichi M., Tanabe M. (2012). KEGG for integration and interpretation of large-scale molecular data sets. Nucleic Acids Res..

